# Supplementary material for: Detailed molecular and epigenetic characterization of the pig IPEC-J2 and chicken SL-29 cell lines
Source: iScience. 2023 Feb 20;26(3):106252. doi: 10.1016/j.isci.2023.106252 (PMC10018572; doi:10.1016/j.isci.2023.106252)
Supplement: Data S2. Complete homer output for identified motifs in Chicken SL-29, related to Tables 5 and 6 — Homer motif analysis results for histone modifications H3K4me1, H3K4me3, H3K27ac, enhancers, and ATAC-seq of chicken SL-29 cell line. Parameters for possible false positives is as mentioned earlier for S5. [file mmc3.zip › Data_S2/S6/Chicken_SL_29/peak_files_H3K27ac/knownResults.html]

peak\_files\_H3K27ac - Homer Known Motif Enrichment Results


# Homer Known Motif Enrichment Results (peak\_files\_H3K27ac)

Homer *de novo* Motif Results  
Gene Ontology Enrichment Results  
Known Motif Enrichment Results (txt file)  
Total Target Sequences = 30160, Total Background Sequences = 30217

|  |  |  |  |  |  |  |  |  |  |  |  |
| --- | --- | --- | --- | --- | --- | --- | --- | --- | --- | --- | --- |
| Rank | Motif | Name | P-value | log P-pvalue | q-value (Benjamini) | # Target Sequences with Motif | % of Targets Sequences with Motif | # Background Sequences with Motif | % of Background Sequences with Motif | Motif File | SVG |
| 1 | T G C A A C T G T A C G A T G C A G T C G A C T T C G A A T C G | ZNF711(Zf)/SHSY5Y-ZNF711-ChIP-Seq(GSE20673)/Homer | 1e-48 | -1.125e+02 | 0.0000 | 6964.0 | 23.09% | 5937.6 | 19.65% | motif file (matrix) | svg |
| 2 | C T G A A C T G A C T G A G T C A G T C A G C T C T A G T A C G | ZFX(Zf)/mES-Zfx-ChIP-Seq(GSE11431)/Homer | 1e-41 | -9.513e+01 | 0.0000 | 4282.0 | 14.20% | 3513.1 | 11.62% | motif file (matrix) | svg |
| 3 | T A G C C G T A C T G A T A C G C G T A A C G T A C T G A C T G A G T C T A C G C T A G G T A C | YY1(Zf)/Promoter/Homer | 1e-25 | -5.878e+01 | 0.0000 | 612.0 | 2.03% | 389.1 | 1.29% | motif file (matrix) | svg |
| 4 | T C G A C T G A T A G C T G A C T C A G T C A G C G T A C G T A T C A G A G C T | ETV1(ETS)/GIST48-ETV1-ChIP-Seq(GSE22441)/Homer | 1e-22 | -5.087e+01 | 0.0000 | 5430.0 | 18.01% | 4809.3 | 15.91% | motif file (matrix) | svg |
| 5 | C T G A T A G C T G A C T C A G C T A G G T C A C G T A T C A G A G C T T C A G | ETV4(ETS)/HepG2-ETV4-ChIP-Seq(ENCODE)/Homer | 1e-21 | -4.885e+01 | 0.0000 | 4693.0 | 15.56% | 4121.8 | 13.64% | motif file (matrix) | svg |
| 6 | T C G A T A G C G T C A A C T G A C T G C G T A C G T A C T A G A G C T T C A G | ERG(ETS)/VCaP-ERG-ChIP-Seq(GSE14097)/Homer | 1e-20 | -4.741e+01 | 0.0000 | 6514.0 | 21.60% | 5871.7 | 19.43% | motif file (matrix) | svg |
| 7 | A T C G A G T C A G T C G A C T A T G C C T G A C T A G A C T G T A C G G T A C C T G A C G A T | AP-2gamma(AP2)/MCF7-TFAP2C-ChIP-Seq(GSE21234)/Homer | 1e-17 | -4.001e+01 | 0.0000 | 5542.0 | 18.38% | 4990.2 | 16.51% | motif file (matrix) | svg |
| 8 | A T G C A G T C C T G A A G T C C G A T A C G T A G T C A G T C A C G T A T C G G A C T A C G T | Etv2(ETS)/ES-ER71-ChIP-Seq(GSE59402)/Homer | 1e-17 | -3.938e+01 | 0.0000 | 3779.0 | 12.53% | 3314.4 | 10.97% | motif file (matrix) | svg |
| 9 | G C T A C G A T C A T G A T G C A G T C A G T C G A C T T A C G T C G A C T A G A C T G T A G C | AP-2alpha(AP2)/Hela-AP2alpha-ChIP-Seq(GSE31477)/Homer | 1e-17 | -3.933e+01 | 0.0000 | 4282.0 | 14.20% | 3791.8 | 12.55% | motif file (matrix) | svg |
| 10 | G A C T T C G A C G A T T A C G C T A G T A C G A C T G A T G C G T A C G T A C | Zac1(Zf)/Neuro2A-Plagl1-ChIP-Seq(GSE75942)/Homer | 1e-15 | -3.678e+01 | 0.0000 | 10435.0 | 34.60% | 9784.8 | 32.38% | motif file (matrix) | svg |
| 11 | T C G A T A G C T G C A A C T G A C T G C G T A C G T A C T A G G A C T T A C G | ETS1(ETS)/Jurkat-ETS1-ChIP-Seq(GSE17954)/Homer | 1e-14 | -3.325e+01 | 0.0000 | 4298.0 | 14.25% | 3848.2 | 12.73% | motif file (matrix) | svg |
| 12 | T C G A T C G A T A G C G T A C T C A G T A C G C G T A C G T A T C A G A G C T | GABPA(ETS)/Jurkat-GABPa-ChIP-Seq(GSE17954)/Homer | 1e-13 | -3.206e+01 | 0.0000 | 3460.0 | 11.47% | 3059.1 | 10.12% | motif file (matrix) | svg |
| 13 | C G T A C G T A C G T A G C A T G C A T T A C G G T A C G A C T A C T G C G T A A T C G A C G T G T A C C G T A A G C T | Bach1(bZIP)/K562-Bach1-ChIP-Seq(GSE31477)/Homer | 1e-13 | -3.198e+01 | 0.0000 | 269.0 | 0.89% | 163.8 | 0.54% | motif file (matrix) | svg |
| 14 | A G T C C T G A A G T C C G A T C A G T G A T C A T G C A C T G A T C G G A C T | Fli1(ETS)/CD8-FLI-ChIP-Seq(GSE20898)/Homer | 1e-13 | -3.105e+01 | 0.0000 | 4661.0 | 15.46% | 4212.6 | 13.94% | motif file (matrix) | svg |
| 15 | C G A T C T G A C G T A C A G T A G T C G A T C G A T C A C T G | Pitx1(Homeobox)/Chicken-Pitx1-ChIP-Seq(GSE38910)/Homer | 1e-13 | -3.035e+01 | 0.0000 | 15449.0 | 51.23% | 14837.0 | 49.09% | motif file (matrix) | svg |
| 16 | C T G A A G T C C G A T A G C T A T G C G T A C A C G T A T C G C A G T G C A T | Elf4(ETS)/BMDM-Elf4-ChIP-Seq(GSE88699)/Homer | 1e-12 | -2.969e+01 | 0.0000 | 3750.0 | 12.43% | 3351.7 | 11.09% | motif file (matrix) | svg |
| 17 | G T C A G C A T A C T G G T A C G A C T A C T G G C T A A T C G C A G T G T A C C G T A A G C T | Nrf2(bZIP)/Lymphoblast-Nrf2-ChIP-Seq(GSE37589)/Homer | 1e-12 | -2.857e+01 | 0.0000 | 230.0 | 0.76% | 138.3 | 0.46% | motif file (matrix) | svg |
| 18 | T A C G G A C T A C T G A C T G C T A G A T G C A G T C A G T C A G T C C T G A | ZNF692(Zf)/HEK293-ZNF692.GFP-ChIP-Seq(GSE58341)/Homer | 1e-11 | -2.579e+01 | 0.0000 | 533.0 | 1.77% | 393.1 | 1.30% | motif file (matrix) | svg |
| 19 | C G T A T A G C T A G C T G C A A C T G C T A G C G T A C G T A T C A G G A C T | EHF(ETS)/LoVo-EHF-ChIP-Seq(GSE49402)/Homer | 1e-11 | -2.537e+01 | 0.0000 | 5182.0 | 17.18% | 4762.2 | 15.76% | motif file (matrix) | svg |
| 20 | G A T C C T G A A G T C C G A T C G A T G A T C A G T C A C T G A T C G A G C T | Elk4(ETS)/Hela-Elk4-ChIP-Seq(GSE31477)/Homer | 1e-10 | -2.525e+01 | 0.0000 | 2218.0 | 7.35% | 1931.4 | 6.39% | motif file (matrix) | svg |
| 21 | C G T A C G T A C G T A G C A T G C A T A C T G G T A C G A C T C T A G G C T A T A C G G A C T T G A C C G T A A G C T | NFE2L2(bZIP)/HepG2-NFE2L2-ChIP-Seq(Encode)/Homer | 1e-10 | -2.446e+01 | 0.0000 | 251.0 | 0.83% | 161.8 | 0.54% | motif file (matrix) | svg |
| 22 | C T G A T G C A T A G C T G A C T A C G T C A G C T G A G C T A T C A G G A C T | ELF1(ETS)/Jurkat-ELF1-ChIP-Seq(SRA014231)/Homer | 1e-9 | -2.259e+01 | 0.0000 | 1870.0 | 6.20% | 1621.1 | 5.36% | motif file (matrix) | svg |
| 23 | C T G A T C A G G T A C G C T A A C T G T G A C G C A T C A T G | SCL(bHLH)/HPC7-Scl-ChIP-Seq(GSE13511)/Homer | 1e-9 | -2.160e+01 | 0.0000 | 20419.0 | 67.71% | 19958.5 | 66.04% | motif file (matrix) | svg |
| 24 | T A C G T C G A C A G T A C T G G C T A A T G C C G A T G T A C C G T A A C T G T A G C C G T A | NF-E2(bZIP)/K562-NFE2-ChIP-Seq(GSE31477)/Homer | 1e-9 | -2.145e+01 | 0.0000 | 259.0 | 0.86% | 173.2 | 0.57% | motif file (matrix) | svg |
| 25 | G A T C T C G A A G T C C G A T C G A T A G T C A T G C A C T G A T C G G A C T | Elk1(ETS)/Hela-Elk1-ChIP-Seq(GSE31477)/Homer | 1e-9 | -2.107e+01 | 0.0000 | 2232.0 | 7.40% | 1971.4 | 6.52% | motif file (matrix) | svg |
| 26 | T C G A A G C T A C G T A C G T A G T C A G T C A C G T A T C G G A C T A T C G | EWS:ERG-fusion(ETS)/CADO\_ES1-EWS:ERG-ChIP-Seq(SRA014231)/Homer | 1e-8 | -2.058e+01 | 0.0000 | 2877.0 | 9.54% | 2587.5 | 8.56% | motif file (matrix) | svg |
| 27 | T G C A C T G A A T G C G T C A A C T G A C T G C G T A C G T A C T A G A G C T | Ets1-distal(ETS)/CD4+-PolII-ChIP-Seq(Barski\_et\_al.)/Homer | 1e-7 | -1.785e+01 | 0.0000 | 1030.0 | 3.42% | 867.6 | 2.87% | motif file (matrix) | svg |
| 28 | G A C T C A G T A G C T C G A T A G T C G A T C A G T C C G T A A T G C T C A G | Rbpj1(?)/Panc1-Rbpj1-ChIP-Seq(GSE47459)/Homer | 1e-7 | -1.750e+01 | 0.0000 | 5436.0 | 18.03% | 5089.6 | 16.84% | motif file (matrix) | svg |
| 29 | T A C G T A C G G T A C A T C G A C T G T A C G T C G A C T G A T C G A A T C G | E2F6(E2F)/Hela-E2F6-ChIP-Seq(GSE31477)/Homer | 1e-7 | -1.745e+01 | 0.0000 | 3595.0 | 11.92% | 3303.0 | 10.93% | motif file (matrix) | svg |
| 30 | T A C G T A C G G T A C A T C G T A C G T A C G G T C A C T G A C G T A G A C T | E2F4(E2F)/K562-E2F4-ChIP-Seq(GSE31477)/Homer | 1e-7 | -1.725e+01 | 0.0000 | 2559.0 | 8.49% | 2310.9 | 7.65% | motif file (matrix) | svg |
| 31 | T G C A T C G A T A G C G T A C T C A G C T A G G T C A G C T A T C A G G A C T | ETS(ETS)/Promoter/Homer | 1e-7 | -1.704e+01 | 0.0000 | 1070.0 | 3.55% | 908.5 | 3.01% | motif file (matrix) | svg |
| 32 | C T A G T C G A C T G A C G T A T A C G G A C T T C A G T C G A G T C A T G C A T A C G A G C T | IRF2(IRF)/Erythroblas-IRF2-ChIP-Seq(GSE36985)/Homer | 1e-7 | -1.643e+01 | 0.0000 | 463.0 | 1.54% | 360.1 | 1.19% | motif file (matrix) | svg |
| 33 | T A G C C G A T T A C G A C T G A G T C A C T G A T C G A T C G C G T A C T G A | E2F1(E2F)/Hela-E2F1-ChIP-Seq(GSE22478)/Homer | 1e-7 | -1.635e+01 | 0.0000 | 1647.0 | 5.46% | 1451.3 | 4.80% | motif file (matrix) | svg |
| 34 | C G T A C A T G C A T G A C T G C T A G T C G A G C A T C G A T A G C T A G T C G A T C G T A C | NFkB-p65(RHD)/GM12787-p65-ChIP-Seq(GSE19485)/Homer | 1e-7 | -1.625e+01 | 0.0000 | 2012.0 | 6.67% | 1797.6 | 5.95% | motif file (matrix) | svg |
| 35 | C T A G C T A G A G T C T C A G A C T G A C G T A C G T C T G A | MYB(HTH)/ERMYB-Myb-ChIPSeq(GSE22095)/Homer | 1e-7 | -1.622e+01 | 0.0000 | 8665.0 | 28.73% | 8276.7 | 27.39% | motif file (matrix) | svg |
| 36 | T G C A C T G A A G T C G T C A A C T G A C T G C G T A C G T A C T G A A G C T | EWS:FLI1-fusion(ETS)/SK\_N\_MC-EWS:FLI1-ChIP-Seq(SRA014231)/Homer | 1e-7 | -1.622e+01 | 0.0000 | 2262.0 | 7.50% | 2035.7 | 6.74% | motif file (matrix) | svg |
| 37 | T C A G C T G A C G T A C G T A T A C G G C A T C T A G C T G A C G T A C G T A T A C G G A C T | IRF1(IRF)/PBMC-IRF1-ChIP-Seq(GSE43036)/Homer | 1e-6 | -1.573e+01 | 0.0000 | 617.0 | 2.05% | 500.2 | 1.66% | motif file (matrix) | svg |
| 38 | G C T A C T G A T C G A A G T C A G T C C T G A A G T C G T C A C T G A T G C A | RUNX1(Runt)/Jurkat-RUNX1-ChIP-Seq(GSE29180)/Homer | 1e-6 | -1.484e+01 | 0.0000 | 3920.0 | 13.00% | 3645.0 | 12.06% | motif file (matrix) | svg |
| 39 | C A G T T C A G A G C T G A C T A C G T A G T C G A T C G A C T C T G A A C T G G A T C C G T A C T G A A G T C G T A C | Rfx6(HTH)/Min6b1-Rfx6.HA-ChIP-Seq(GSE62844)/Homer | 1e-6 | -1.426e+01 | 0.0000 | 5045.0 | 16.73% | 4746.2 | 15.70% | motif file (matrix) | svg |
| 40 | T C A G C T A G C T A G T G C A C G A T C G A T C G T A C T A G | GSC(Homeobox)/FrogEmbryos-GSC-ChIP-Seq(DRA000576)/Homer | 1e-6 | -1.422e+01 | 0.0000 | 4126.0 | 13.68% | 3851.2 | 12.74% | motif file (matrix) | svg |
| 41 | A G T C G A T C A G T C C G T A A T C G C A G T A G T C G T A C C T G A A C T G T C A G A G C T A G C T A G C T A G C T | PRDM15(Zf)/ESC-Prdm15-ChIP-Seq(GSE73694)/Homer | 1e-5 | -1.303e+01 | 0.0000 | 3792.0 | 12.57% | 3540.8 | 11.72% | motif file (matrix) | svg |
| 42 | C G A T T A C G T G A C G A C T C A T G C G T A T A C G A C G T G T A C C T G A | Bach2(bZIP)/OCILy7-Bach2-ChIP-Seq(GSE44420)/Homer | 1e-5 | -1.299e+01 | 0.0000 | 660.0 | 2.19% | 552.0 | 1.83% | motif file (matrix) | svg |
| 43 | T G A C C T A G T C A G G T C A C G T A T C A G C G A T T C A G T C G A T G C A C T G A T A G C | PU.1-IRF(ETS:IRF)/Bcell-PU.1-ChIP-Seq(GSE21512)/Homer | 1e-5 | -1.294e+01 | 0.0000 | 5625.0 | 18.65% | 5331.3 | 17.64% | motif file (matrix) | svg |
| 44 | C T G A T A C G G C A T A G C T A G C T A G T C T C G A A C T G C A G T A G C T A G C T G A T C | IRF3(IRF)/BMDM-Irf3-ChIP-Seq(GSE67343)/Homer | 1e-5 | -1.277e+01 | 0.0000 | 1358.0 | 4.50% | 1203.7 | 3.98% | motif file (matrix) | svg |
| 45 | C T G A A T C G A G C T A G C T A C G T T A G C C T G A T A C G C G A T A C G T G A C T A G T C | ISRE(IRF)/ThioMac-LPS-Expression(GSE23622)/Homer | 1e-5 | -1.270e+01 | 0.0000 | 246.0 | 0.82% | 182.5 | 0.60% | motif file (matrix) | svg |
| 46 | A C T G C G T A A C T G A T G C T G A C G A T C A T C G T G C A A C T G A G T C | ZNF519(Zf)/HEK293-ZNF519.GFP-ChIP-Seq(GSE58341)/Homer | 1e-5 | -1.261e+01 | 0.0000 | 1307.0 | 4.33% | 1156.9 | 3.83% | motif file (matrix) | svg |
| 47 | T C G A T G C A A C T G G T C A A G T C A G T C A G T C A G T C A G C T T G A C | LRF(Zf)/Erythroblasts-ZBTB7A-ChIP-Seq(GSE74977)/Homer | 1e-5 | -1.261e+01 | 0.0000 | 6206.0 | 20.58% | 5906.3 | 19.54% | motif file (matrix) | svg |
| 48 | G C T A T A G C A G C T A T C G G T C A C G T A G C T A A T G C G A T C C T G A | IRF4(IRF)/GM12878-IRF4-ChIP-Seq(GSE32465)/Homer | 1e-5 | -1.247e+01 | 0.0000 | 1931.0 | 6.40% | 1750.5 | 5.79% | motif file (matrix) | svg |
| 49 | C A T G A C G T A G T C G A T C G A T C G A T C G C T A C T A G C T A G C T A G T C A G T C G A | EBF1(EBF)/Near-E2A-ChIP-Seq(GSE21512)/Homer | 1e-5 | -1.229e+01 | 0.0000 | 4163.0 | 13.80% | 3910.3 | 12.94% | motif file (matrix) | svg |
| 50 | G C T A A G T C T A C G T G C A A T C G T C A G G C T A T C G A T C A G A G C T | ELF5(ETS)/T47D-ELF5-ChIP-Seq(GSE30407)/Homer | 1e-5 | -1.215e+01 | 0.0000 | 2782.0 | 9.23% | 2571.3 | 8.51% | motif file (matrix) | svg |
| 51 | C G T A T G A C T A G C T G C A A C T G A C T G C G T A C G T A T C A G G A C T | ELF3(ETS)/PDAC-ELF3-ChIP-Seq(GSE64557)/Homer | 1e-5 | -1.161e+01 | 0.0001 | 2774.0 | 9.20% | 2569.3 | 8.50% | motif file (matrix) | svg |
| 52 | A T G C T C G A T A C G A C G T A T G C A G T C A C G T A G T C A G T C G A T C | Znf263(Zf)/K562-Znf263-ChIP-Seq(GSE31477)/Homer | 1e-5 | -1.156e+01 | 0.0001 | 9435.0 | 31.29% | 9112.1 | 30.15% | motif file (matrix) | svg |
| 53 | T C A G T C A G G C T A C G T A T A C G G A C T T C A G T C G A C T G A C G T A T A C G G A C T | IRF8(IRF)/BMDM-IRF8-ChIP-Seq(GSE77884)/Homer | 1e-4 | -1.144e+01 | 0.0001 | 1333.0 | 4.42% | 1189.2 | 3.93% | motif file (matrix) | svg |
| 54 | C T G A A T G C C G T A A C G T A G T C A G T C A C G T A C T G A T C G G C A T | SPDEF(ETS)/VCaP-SPDEF-ChIP-Seq(SRA014231)/Homer | 1e-4 | -1.122e+01 | 0.0001 | 3942.0 | 13.07% | 3708.8 | 12.27% | motif file (matrix) | svg |
| 55 | C T G A T C A G A C T G C A G T A G T C G A C T A G T C A C G T C G T A G C T A A T G C G A T C | PRDM14(Zf)/H1-PRDM14-ChIP-Seq(GSE22767)/Homer | 1e-4 | -1.088e+01 | 0.0001 | 821.0 | 2.72% | 711.8 | 2.36% | motif file (matrix) | svg |
| 56 | A T C G G A T C G C A T C G T A G T C A A C G T A T G C A G T C | CRX(Homeobox)/Retina-Crx-ChIP-Seq(GSE20012)/Homer | 1e-4 | -1.084e+01 | 0.0002 | 8311.0 | 27.56% | 8011.0 | 26.51% | motif file (matrix) | svg |
| 57 | C G T A C T G A C T A G C G T A C G T A A G T C C G T A C A G T G C A T G T C A C G A T A C T G A C G T G C A T G A T C | PGR(NR)/EndoStromal-PGR-ChIP-Seq(GSE69539)/Homer | 1e-4 | -1.068e+01 | 0.0002 | 999.0 | 3.31% | 879.0 | 2.91% | motif file (matrix) | svg |
| 58 | A T G C T C G A A G T C A G C T A C G T G T A C A G T C G C T A C T A G C A T G G T C A C T G A T C A G A G T C | Stat3+il21(Stat)/CD4-Stat3-ChIP-Seq(GSE19198)/Homer | 1e-4 | -1.015e+01 | 0.0003 | 2712.0 | 8.99% | 2525.6 | 8.36% | motif file (matrix) | svg |
| 59 | A C T G A C G T C A T G A T C G A T C G T G A C A C T G A T C G A T C G T G C A C T G A C G T A | E2F3(E2F)/MEF-E2F3-ChIP-Seq(GSE71376)/Homer | 1e-4 | -1.011e+01 | 0.0003 | 3799.0 | 12.60% | 3583.0 | 11.86% | motif file (matrix) | svg |
| 60 | A C T G G A T C G A C T A C T G A C G T C A T G A C T G A C G T A G C T C G A T | RUNX-AML(Runt)/CD4+-PolII-ChIP-Seq(Barski\_et\_al.)/Homer | 1e-4 | -9.663e+00 | 0.0005 | 2906.0 | 9.64% | 2719.5 | 9.00% | motif file (matrix) | svg |
| 61 | T A C G A T C G T A G C G A T C A C T G A C G T A G T C A C G T C T A G A T C G | Smad4(MAD)/ESC-SMAD4-ChIP-Seq(GSE29422)/Homer | 1e-4 | -9.444e+00 | 0.0006 | 5918.0 | 19.62% | 5672.1 | 18.77% | motif file (matrix) | svg |
| 62 | T A C G C T G A C A T G G A T C G T A C G C A T T C A G T A C G A G C T G T C A G A T C G C A T T A C G C G T A C T A G G A T C G A T C C G A T A C T G T C A G | ZNF322(Zf)/HEK293-ZNF322.GFP-ChIP-Seq(GSE58341)/Homer | 1e-4 | -9.390e+00 | 0.0006 | 980.0 | 3.25% | 870.9 | 2.88% | motif file (matrix) | svg |
| 63 | A G T C G C A T C G T A C G T A G T A C A C G T A C T G G A T C G A T C T C G A | BMYB(HTH)/Hela-BMYB-ChIP-Seq(GSE27030)/Homer | 1e-4 | -9.379e+00 | 0.0006 | 7227.0 | 23.96% | 6965.9 | 23.05% | motif file (matrix) | svg |
| 64 | C T G A A G C T A C G T A C G T A G T C G A C T G A C T C T G A C T G A C T A G C G T A C G T A | STAT6(Stat)/CD4-Stat6-ChIP-Seq(GSE22104)/Homer | 1e-4 | -9.262e+00 | 0.0007 | 2272.0 | 7.53% | 2109.6 | 6.98% | motif file (matrix) | svg |
| 65 | T C G A T C G A T A C G G A T C G T C A G T A C C G A T A G C T G T C A T G C A | Nkx3.1(Homeobox)/LNCaP-Nkx3.1-ChIP-Seq(GSE28264)/Homer | 1e-3 | -8.983e+00 | 0.0008 | 9417.0 | 31.23% | 9143.7 | 30.26% | motif file (matrix) | svg |
| 66 | C A G T G A C T C G T A G C T A G T A C G A T C G T A C G A C T A G C T A C G T T G A C C G T A C A G T A C G T A T G C | ZNF652/HepG2-ZNF652.Flag-ChIP-Seq(Encode)/Homer | 1e-3 | -8.853e+00 | 0.0010 | 941.0 | 3.12% | 837.8 | 2.77% | motif file (matrix) | svg |
| 67 | A G T C G A C T C A G T G T A C A G T C A T C G T C A G A C T G G T C A C G T A | Stat3(Stat)/mES-Stat3-ChIP-Seq(GSE11431)/Homer | 1e-3 | -8.505e+00 | 0.0013 | 1727.0 | 5.73% | 1591.9 | 5.27% | motif file (matrix) | svg |
| 68 | T A G C G C A T A G T C G A T C A T G C G A C T C T A G A C T G A C T G C T G A A C T G C T A G A G T C T G A C C G A T | GLIS3(Zf)/Thyroid-Glis3.GFP-ChIP-Seq(GSE103297)/Homer | 1e-3 | -8.467e+00 | 0.0014 | 6874.0 | 22.79% | 6633.3 | 21.95% | motif file (matrix) | svg |
| 69 | G A C T C T A G C T A G A G T C T G C A A C T G A C G T A C G T C T A G T C A G | AMYB(HTH)/Testes-AMYB-ChIP-Seq(GSE44588)/Homer | 1e-3 | -8.288e+00 | 0.0016 | 7094.0 | 23.52% | 6854.8 | 22.68% | motif file (matrix) | svg |
| 70 | T A C G A T C G G A T C G T C A C T G A G C A T C G A T G C T A T C G A G C T A | Unknown(Homeobox)/Limb-p300-ChIP-Seq/Homer | 1e-3 | -8.287e+00 | 0.0016 | 2807.0 | 9.31% | 2640.6 | 8.74% | motif file (matrix) | svg |
| 71 | C G T A C T G A A C T G A G C T A G T C G A T C G A T C G C A T C T G A C T A G C T A G T A C G T C G A T G C A G C A T | EBF2(EBF)/BrownAdipose-EBF2-ChIP-Seq(GSE97114)/Homer | 1e-3 | -8.099e+00 | 0.0019 | 3107.0 | 10.30% | 2935.1 | 9.71% | motif file (matrix) | svg |
| 72 | C A T G A G T C A G C T C G T A C G A T C G A T G C A T G C A T C G A T C T G A C A T G T G A C | Mef2d(MADS)/Retina-Mef2d-ChIP-Seq(GSE61391)/Homer | 1e-3 | -7.971e+00 | 0.0021 | 784.0 | 2.60% | 695.0 | 2.30% | motif file (matrix) | svg |
| 73 | C T A G T C G A A C G T A C T G C G T A A T G C A C G T G T A C C G T A A G C T G A T C G T A C | Atf3(bZIP)/GBM-ATF3-ChIP-Seq(GSE33912)/Homer | 1e-3 | -7.912e+00 | 0.0022 | 2126.0 | 7.05% | 1983.1 | 6.56% | motif file (matrix) | svg |
| 74 | A G T C G T A C A G C T C T A G A G T C C G A T A C T G C G T A A C T G G T C A | Zic(Zf)/Cerebellum-ZIC1.2-ChIP-Seq(GSE60731)/Homer | 1e-3 | -7.681e+00 | 0.0027 | 4889.0 | 16.21% | 4689.1 | 15.52% | motif file (matrix) | svg |
| 75 | C T G A T C G A C G T A A T G C C G T A C G T A C G A T C T A G T C A G G A T C | Sox15(HMG)/CPA-Sox15-ChIP-Seq(GSE62909)/Homer | 1e-3 | -7.635e+00 | 0.0028 | 4054.0 | 13.44% | 3869.8 | 12.80% | motif file (matrix) | svg |
| 76 | T C G A A C G T C A T G G C T A T A G C C G A T G T A C G C T A A C G T A T G C | AP-1(bZIP)/ThioMac-PU.1-ChIP-Seq(GSE21512)/Homer | 1e-3 | -7.478e+00 | 0.0033 | 2377.0 | 7.88% | 2232.3 | 7.39% | motif file (matrix) | svg |
| 77 | A T G C A G T C G T A C A G C T T C G A C T A G G A T C C T G A G T C A A G T C G C T A T C A G | Rfx5(HTH)/GM12878-Rfx5-ChIP-Seq(GSE31477)/Homer | 1e-3 | -7.450e+00 | 0.0033 | 1062.0 | 3.52% | 963.5 | 3.19% | motif file (matrix) | svg |
| 78 | C G T A T A C G T C G A A C T G A C T G C G T A C G T A T A C G A G C T T A C G | PU.1(ETS)/ThioMac-PU.1-ChIP-Seq(GSE21512)/Homer | 1e-3 | -7.106e+00 | 0.0046 | 1635.0 | 5.42% | 1517.7 | 5.02% | motif file (matrix) | svg |
| 79 | T C A G A G C T A C G T A C G T G T A C G A T C C G T A C T A G C A T G G T C A C G T A T C G A | STAT4(Stat)/CD4-Stat4-ChIP-Seq(GSE22104)/Homer | 1e-3 | -6.911e+00 | 0.0055 | 3776.0 | 12.52% | 3608.2 | 11.94% | motif file (matrix) | svg |
| 80 | G A C T C G A T T C A G G A T C G A C T A G C T A G C T A G T C G A T C C G T A C T A G C T A G T C G A T C G A C T G A | Bcl6(Zf)/Liver-Bcl6-ChIP-Seq(GSE31578)/Homer | 1e-2 | -6.762e+00 | 0.0064 | 4797.0 | 15.91% | 4615.3 | 15.27% | motif file (matrix) | svg |
| 81 | G A C T A G C T G T A C G A C T C T G A A C T G G T C A C T G A A T G C T A C G G A C T A C G T A G T C G A C T C T G A | HRE(HSF)/Striatum-HSF1-ChIP-Seq(GSE38000)/Homer | 1e-2 | -6.722e+00 | 0.0065 | 552.0 | 1.83% | 485.1 | 1.61% | motif file (matrix) | svg |
| 82 | T G A C G C T A T C G A T G C A A G T C A G T C C G T A A G T C C G T A C T G A G C T A G T A C | RUNX2(Runt)/PCa-RUNX2-ChIP-Seq(GSE33889)/Homer | 1e-2 | -6.687e+00 | 0.0067 | 3044.0 | 10.09% | 2894.3 | 9.58% | motif file (matrix) | svg |
| 83 | C G T A C T A G T C A G T C A G A G T C A T G C A G T C G C A T A G C T A C G T A T C G C G A T | Sox9(HMG)/Limb-SOX9-ChIP-Seq(GSE73225)/Homer | 1e-2 | -6.659e+00 | 0.0068 | 3161.0 | 10.48% | 3009.1 | 9.96% | motif file (matrix) | svg |
| 84 | C A G T C G A T G C A T G C A T G T C A A G C T C A T G C T A G A T G C G T A C | Hoxa11(Homeobox)/ChickenMSG-Hoxa11.Flag-ChIP-Seq(GSE86088)/Homer | 1e-2 | -6.607e+00 | 0.0071 | 9786.0 | 32.45% | 9563.8 | 31.65% | motif file (matrix) | svg |
| 85 | C G A T A C G T A C G T A C G T C G T A A G C T C A G T C T A G A T C G A C T G | HOXB13(Homeobox)/ProstateTumor-HOXB13-ChIP-Seq(GSE56288)/Homer | 1e-2 | -6.561e+00 | 0.0073 | 4313.0 | 14.30% | 4142.2 | 13.71% | motif file (matrix) | svg |
| 86 | T A G C G C T A T C G A C T G A A G T C A G T C C T G A A G T C C G T A C T A G | RUNX(Runt)/HPC7-Runx1-ChIP-Seq(GSE22178)/Homer | 1e-2 | -6.521e+00 | 0.0075 | 2489.0 | 8.25% | 2355.0 | 7.79% | motif file (matrix) | svg |
| 87 | A G C T C T A G T G A C C G T A A C G T C G A T A G T C A G T C C T G A C A T G | TEAD3(TEA)/HepG2-TEAD3-ChIP-Seq(Encode)/Homer | 1e-2 | -6.417e+00 | 0.0083 | 3858.0 | 12.79% | 3697.1 | 12.23% | motif file (matrix) | svg |
| 88 | C A G T T C A G G A T C A C T G A C G T C T A G A C T G A C T G G A C T C T A G | Egr1(Zf)/K562-Egr1-ChIP-Seq(GSE32465)/Homer | 1e-2 | -6.398e+00 | 0.0083 | 3219.0 | 10.67% | 3070.8 | 10.16% | motif file (matrix) | svg |
| 89 | C A T G A G T C G A C T C G T A C G A T G C A T G A C T G C A T C G A T C T A G C A T G T G A C | Mef2b(MADS)/HEK293-Mef2b.V5-ChIP-Seq(GSE67450)/Homer | 1e-2 | -6.196e+00 | 0.0101 | 3234.0 | 10.72% | 3088.3 | 10.22% | motif file (matrix) | svg |
| 90 | A G T C C G A T C T G A C G T A A C G T C A G T T C A G T G A C | Isl1(Homeobox)/Neuron-Isl1-ChIP-Seq(GSE31456)/Homer | 1e-2 | -6.150e+00 | 0.0104 | 7489.0 | 24.83% | 7291.0 | 24.13% | motif file (matrix) | svg |
| 91 | A G T C A G T C C G A T A C G T A C G T A C T G A C G T A G C T A G T C A G T C | Sox4(HMG)/proB-Sox4-ChIP-Seq(GSE50066)/Homer | 1e-2 | -6.128e+00 | 0.0105 | 3571.0 | 11.84% | 3420.8 | 11.32% | motif file (matrix) | svg |
| 92 | C A T G A G T C C T G A T G A C A T C G G A C T G T C A A G T C T A G C G A T C | HIF2a(bHLH)/785\_O-HIF2a-ChIP-Seq(GSE34871)/Homer | 1e-2 | -6.018e+00 | 0.0116 | 2010.0 | 6.67% | 1894.7 | 6.27% | motif file (matrix) | svg |
| 93 | C G A T C T A G A C G T A C G T A C G T C G T A A G C T C G A T A G C T C G T A C T A G T A G C | FoxD3(forkhead)/ZebrafishEmbryo-Foxd3.biotin-ChIP-seq(GSE106676)/Homer | 1e-2 | -5.907e+00 | 0.0129 | 3745.0 | 12.42% | 3595.7 | 11.90% | motif file (matrix) | svg |
| 94 | C T G A C T A G T C G A C G T A A T G C C G T A A T C G C G A T T A G C G C A T A T C G G C A T A G C T G A T C G A C T A G C T | ARE(NR)/LNCAP-AR-ChIP-Seq(GSE27824)/Homer | 1e-2 | -5.875e+00 | 0.0131 | 1074.0 | 3.56% | 989.6 | 3.27% | motif file (matrix) | svg |
| 95 | A T G C G A T C G A C T A G C T C G A T C G A T G T C A C G A T T C G A A T C G T A G C T A G C | TATA-Box(TBP)/Promoter/Homer | 1e-2 | -5.780e+00 | 0.0143 | 5057.0 | 16.77% | 4891.0 | 16.18% | motif file (matrix) | svg |
| 96 | G A C T C A G T G A T C G A T C A C G T G A T C C T G A T A C G C G T A G T C A | STAT6(Stat)/Macrophage-Stat6-ChIP-Seq(GSE38377)/Homer | 1e-2 | -5.779e+00 | 0.0143 | 2265.0 | 7.51% | 2146.6 | 7.10% | motif file (matrix) | svg |
| 97 | T C A G C T A G C T A G C T A G T C A G T C G A C T G A C G A T A G T C G A T C A G T C T G A C | NFkB-p50,p52(RHD)/Monocyte-p50-ChIP-Chip(Schreiber\_et\_al.)/Homer | 1e-2 | -5.577e+00 | 0.0172 | 688.0 | 2.28% | 622.8 | 2.06% | motif file (matrix) | svg |
| 98 | C T A G C T A G C G T A C G T A T A C G C G A T C T A G C T G A C T G A C G T A T A C G G A C T | PU.1:IRF8(ETS:IRF)/pDC-Irf8-ChIP-Seq(GSE66899)/Homer | 1e-2 | -5.546e+00 | 0.0175 | 759.0 | 2.52% | 690.4 | 2.28% | motif file (matrix) | svg |
| 99 | G T A C C G T A C G T A T A C G G C A T G T A C C G T A C A T G A G T C C G T A C G T A C G A T G C A T G C A T G A C T | MafF(bZIP)/HepG2-MafF-ChIP-Seq(GSE31477)/Homer | 1e-2 | -5.460e+00 | 0.0189 | 1255.0 | 4.16% | 1168.1 | 3.87% | motif file (matrix) | svg |
| 100 | A C T G C A T G T C G A A C G T A C T G C G T A A T C G A C G T G T A C C G T A G A C T A G T C | Fos(bZIP)/TSC-Fos-ChIP-Seq(GSE110950)/Homer | 1e-2 | -5.450e+00 | 0.0189 | 1845.0 | 6.12% | 1741.6 | 5.76% | motif file (matrix) | svg |
| 101 | T A G C C G A T A C G T A G C T A G C T A G T C A T G C A G T C A C T G A T G C A T G C G C T A | E2F7(E2F)/Hela-E2F7-ChIP-Seq(GSE32673)/Homer | 1e-2 | -5.404e+00 | 0.0196 | 613.0 | 2.03% | 552.0 | 1.83% | motif file (matrix) | svg |
| 102 | A G T C G A T C G C T A C G A T A C G T T A C G G C A T C T G A G A C T A C T G A G T C G C T A C T G A T C G A C A G T | Oct4:Sox17(POU,Homeobox,HMG)/F9-Sox17-ChIP-Seq(GSE44553)/Homer | 1e-2 | -5.391e+00 | 0.0197 | 595.0 | 1.97% | 536.0 | 1.77% | motif file (matrix) | svg |
| 103 | T C G A G C A T A C G T C T A G G T A C T C G A G C A T T G A C T C G A A C G T | Chop(bZIP)/MEF-Chop-ChIP-Seq(GSE35681)/Homer | 1e-2 | -5.389e+00 | 0.0197 | 991.0 | 3.29% | 915.0 | 3.03% | motif file (matrix) | svg |
| 104 | T C A G T A C G G A T C G T A C T C G A C G A T C T G A C G T A G C T A C G T A | Hoxd11(Homeobox)/ChickenMSG-Hoxd11.Flag-ChIP-Seq(GSE86088)/Homer | 1e-2 | -5.355e+00 | 0.0200 | 9977.0 | 33.08% | 9786.8 | 32.38% | motif file (matrix) | svg |
| 105 | T G A C G T C A A G T C G C T A C T A G A G T C C T G A C A T G A C T G C T A G T A C G T C A G | Zic2(Zf)/ESC-Zic2-ChIP-Seq(SRP197560)/Homer | 1e-2 | -5.214e+00 | 0.0228 | 3682.0 | 12.21% | 3546.3 | 11.73% | motif file (matrix) | svg |
| 106 | A C T G C T A G T C G A C G A T C A T G G C T A A T C G C G A T G T A C G C T A A G C T G T A C | Fra1(bZIP)/BT549-Fra1-ChIP-Seq(GSE46166)/Homer | 1e-2 | -5.174e+00 | 0.0235 | 1720.0 | 5.70% | 1623.3 | 5.37% | motif file (matrix) | svg |
| 107 | C T A G T C G A G C A T C A T G G C T A T A G C C G A T G T A C C T G A A G C T | JunB(bZIP)/DendriticCells-Junb-ChIP-Seq(GSE36099)/Homer | 1e-2 | -5.125e+00 | 0.0245 | 1631.0 | 5.41% | 1537.3 | 5.09% | motif file (matrix) | svg |
| 108 | C A T G A C T G C T A G T C G A T C G A T C G A T C G A T C A G T C A G T C A G T G A C T G A C C G T A A C T G T G C A C G A T A C T G | RBPJ:Ebox(?,bHLH)/Panc1-Rbpj1-ChIP-Seq(GSE47459)/Homer | 1e-2 | -5.042e+00 | 0.0263 | 1348.0 | 4.47% | 1263.7 | 4.18% | motif file (matrix) | svg |
| 109 | T A G C G A T C G T A C C T G A G C A T C T G A C G T A T G C A C G T A G A T C | Hoxa13(Homeobox)/ChickenMSG-Hoxa13.Flag-ChIP-Seq(GSE86088)/Homer | 1e-2 | -4.957e+00 | 0.0284 | 10096.0 | 33.48% | 9916.7 | 32.81% | motif file (matrix) | svg |
| 110 | A T G C A G T C G A T C C G T A A C G T A C G T A C T G A C G T A G C T G A T C | Sox2(HMG)/mES-Sox2-ChIP-Seq(GSE11431)/Homer | 1e-2 | -4.945e+00 | 0.0285 | 3432.0 | 11.38% | 3305.0 | 10.94% | motif file (matrix) | svg |
| 111 | T A G C T A G C G C A T C A T G A C T G G C T A C G T A A C G T A C T G G A T C | TEAD4(TEA)/Tropoblast-Tead4-ChIP-Seq(GSE37350)/Homer | 1e-2 | -4.875e+00 | 0.0303 | 2838.0 | 9.41% | 2722.1 | 9.01% | motif file (matrix) | svg |
| 112 | A G T C A C G T A C T G A G C T A C G T A C G T G T C A A G T C | Foxo1(Forkhead)/RAW-Foxo1-ChIP-Seq(Fan\_et\_al.)/Homer | 1e-2 | -4.860e+00 | 0.0304 | 7568.0 | 25.10% | 7402.6 | 24.49% | motif file (matrix) | svg |
| 113 | A C G T A G T C A G T C C G A T A C G T A C G T A C T G A C G T A T G C G A C T A C T G T A C G | Sox21(HMG)/ESC-SOX21-ChIP-Seq(GSE110505)/Homer | 1e-2 | -4.855e+00 | 0.0304 | 7272.0 | 24.11% | 7108.4 | 23.52% | motif file (matrix) | svg |
| 114 | C T A G T C G A C G A T C T A G G C A T C A G T C T A G G A T C C G T A G T C A | CEBP:AP1(bZIP)/ThioMac-CEBPb-ChIP-Seq(GSE21512)/Homer | 1e-2 | -4.783e+00 | 0.0323 | 3444.0 | 11.42% | 3320.9 | 10.99% | motif file (matrix) | svg |
| 115 | C T A G C G T A G T C A C G T A A G T C G A T C A G C T C T A G C G T A A C G T G T C A G A T C | Six2(Homeobox)/NephronProgenitor-Six2-ChIP-Seq(GSE39837)/Homer | 1e-2 | -4.733e+00 | 0.0337 | 3376.0 | 11.19% | 3254.8 | 10.77% | motif file (matrix) | svg |
| 116 | T G A C A G T C C T G A T G A C C G T A A C G T A C G T A G T C A G T C C G T A | TEAD1(TEAD)/HepG2-TEAD1-ChIP-Seq(Encode)/Homer | 1e-2 | -4.730e+00 | 0.0337 | 3248.0 | 10.77% | 3128.7 | 10.35% | motif file (matrix) | svg |
